# Perturbation of semaphorin and VEGF signaling in ACDMPV lungs due to *FOXF1* deficiency

**DOI:** 10.1186/s12931-021-01797-7

**Published:** 2021-07-27

**Authors:** Justyna A. Karolak, Tomasz Gambin, Przemyslaw Szafranski, Rebecca L. Maywald, Edwina Popek, Jason D. Heaney, Paweł Stankiewicz

**Affiliations:** 1 Department of Molecular and Human Genetics, Baylor College of Medicine, One Baylor Plaza, Rm ABBR-R809, Houston, TX 77030 USA; 2 Chair and Department of Genetics and Pharmaceutical Microbiology, Poznan University of Medical Sciences, 60-781 Poznań, Poland; 3 Institute of Computer Science, Warsaw University of Technology, 00-665 Warsaw, Poland; 4 Department of Pathology and Immunology, Baylor College of Medicine, Houston, TX 77030 USA

**Keywords:** Plexins, Neuropilins, Vascular development, Lung branching, Lethal lung developmental disorders

## Abstract

**Background:**

Alveolar capillary dysplasia with misalignment of pulmonary veins (ACDMPV) is a rare lethal congenital lung disorder in neonates characterized by severe progressive respiratory failure and refractory pulmonary hypertension, resulting from underdevelopment of the peripheral pulmonary tree. Causative heterozygous single nucleotide variants (SNVs) or copy-number variant (CNV) deletions involving *FOXF1* or its distant lung-specific enhancer on chromosome 16q24.1 have been identified in 80–90% of ACDMPV patients. *FOXF1* maps closely to and regulates the oppositely oriented *FENDRR*, with which it also shares regulatory elements.

**Methods:**

To better understand the transcriptional networks downstream of FOXF1 that are relevant for lung organogenesis, using RNA-seq, we have examined lung transcriptomes in 12 histopathologically verified ACDMPV patients with or without pathogenic variants in the *FOXF1* locus and analyzed gene expression profile in *FENDRR*-depleted fetal lung fibroblasts, IMR-90.

**Results:**

RNA-seq analyses in ACDMPV neonates revealed changes in the expression of several genes, including semaphorins (*SEMA*s), neuropilin 1 (*NRP1*), and plexins (*PLXN*s), essential for both epithelial branching and vascular patterning. In addition, we have found deregulation of the vascular endothelial growth factor (VEGF) signaling that also controls pulmonary vasculogenesis and a lung-specific endothelial gene *TMEM100* known to be essential in vascular morphogenesis. Interestingly, we have observed a substantial difference in gene expression profiles between the ACDMPV samples with different types of *FOXF1* defect. Moreover, partial overlap between transcriptome profiles of ACDMPV lungs with *FOXF1* SNVs and *FENDRR*-depleted IMR-90 cells suggests contribution of *FENDRR* to ACDMPV etiology.

**Conclusions:**

Our transcriptomic data imply potential crosstalk between several lung developmental pathways, including interactions between FOXF1-SHH and SEMA-NRP or VEGF/VEGFR2 signaling, and provide further insight into complexity of lung organogenesis in humans.

**Supplementary Information:**

The online version contains supplementary material available at 10.1186/s12931-021-01797-7.

## Background

Alveolar Capillary Dysplasia with Misalignment of Pulmonary Veins (ACDMPV, MIM #265380) is a rarely diagnosed lethal lung developmental disorder (LLDD) in neonates [1–6]. Affected newborns manifest severe progressive hypoxemic respiratory failure and pulmonary arterial hypertension (PAH), refractory to treatment. Most ACDMPV infants show disease symptoms within the first 24–48 h of life and die usually within the first month of life. Very rarely, the course of ACDMPV is milder and patients survive beyond the neonatal period [7–15]. Histopathological findings in ACDMPV include reduced number of pulmonary capillaries, majority of which do not make contact with the alveolar epithelium, thickening of intra-alveolar septa and reduced alveolarization, medial hypertrophy of small peripheral pulmonary arteries and arterioles, and the presence of vascular anastomoses. In addition, most patients with ACDMPV often manifest anomalies of the cardiovascular, gastrointestinal, and genitourinary systems [3–5, 16].

In approximately 80–90% of ACDMPV patients, heterozygous single nucleotide variants (SNVs) or copy-number variant (CNV) deletions involving *FOXF1* (MIM #601089) and/or its non-coding regulatory regions on chromosome 16q24.1 have been found [16–18]. *FOXF1* encodes a forkhead-box (FOX) family transcription factor [19, 20]. In developing lungs, *FOXF1* is expressed in peripheral lung mesenchyme where it mediates sonic hedgehog (SHH) signaling from epithelial cells of branching tubular structures [21–29]. *FOXF1* expression in the lungs is controlled by a distant lung-specific enhancer region located ~ 270 kb upstream to the gene (chr16:86,178,434–86,238,313; hg38) [16, 18, 30–32].

*FOXF1* maps 1.7 kb from the oppositely oriented long noncoding RNA (lncRNA) gene, *FENDRR*, with which it shares regulatory regions that include the promoters and the enhancer. Most recently, *FENDRR* expression was found to be regulated both in *cis* by the *FOXF1* distant lung-specific enhancer and in *trans* by FOXF1, implying involvement of *FENDRR* in *FOXF1*-linked diseases including ACDMPV [33].

Here, we have examined lung transcriptomes of ACDMPV patients with or without pathogenic variants in the *FOXF1* locus on chromosome 16q24.1 to identify gene expression profiles specific for ACDMPV. Using comparative analyses and an over-representation approach, we have investigated deregulated pathways to elucidate potential crosstalk between *FOXF1* and other genes required for normal lung development in humans. We have compared also transcriptome profiles of ACDMPV lungs with transcriptomes of *FENDRR*-depleted fetal lung fibroblasts, IMR-90, to further consider the possibility of the contribution of* FENDRR* deficiency to etiology of ACDMPV.

## Methods

### Patients and samples

Peripheral blood, saliva, and/or frozen or formalin-fixed paraffin-embedded (FFPE) lung biopsy or autopsy samples were obtained from 12 unrelated patients with histopathologically verified ACDMPV (Additional file 1, Additional file 2). Three normal lung tissues from age-matched individuals were used as controls. The study protocol was approved by the Institutional Review Board for Human Subject Research at Baylor College of Medicine (BCM; H-8712).

Human fetal lung fibroblasts IMR-90 (ATCC, Manassas, VA) were cultured at 37 °C in 5% CO_2_ in Eagle's Minimal Essential Medium (ATCC), supplemented with 2 mM l-glutamine, 1 mM sodium pyruvate, and 10% fetal bovine serum.

### DNA and RNA extraction

DNA was isolated from blood and saliva using Gentra Purgene Blood Kit (Qiagen, Germantown, MD), or from frozen lung tissue using DNeasy Blood and Tissue Kit (Qiagen). RNA from frozen lung or FFPE lung tissues was extracted using miRNeasy Mini Kit (Qiagen) or Quick-RNA FFPE Kit (Zymo Research, Irvine, CA), respectively. DNA and RNA from cultured IMR-90 cells was isolated using DNaesy Blood and Tissue Kit (Qiagen) and miRNeasy Mini Kit (Qiagen), respectively.

### Genomic analyses

SNVs were identified by Sanger sequencing of PCR fragments amplified from genomic DNA. Pathogenic CNV deletions were identified and mapped by array comparative genomic hybridization (array CGH), using high resolution custom-designed 4 × 180 K oligo arrays (Agilent Technologies, Santa Clara, CA), followed by Sanger sequencing of long-range-PCR-amplified deletion junctions. Parental origin of the deletion-bearing chromosome 16q24.1 was determined using informative microsatellites or single nucleotide polymorphisms (SNPs).

Based on genomic findings, the ACDMPV patients were divided into five groups, four of which included cases featuring *FOXF1*-related defects at chromosome 16q24.1: CNV deletions involving *FOXF1* enhancer (n = 4; group 1), *FOXF1* and its enhancer (*n* = 3; group 2) or* FOXF1* (*n* = 2; group 3), as well as *FOXF1* mutation (n = 2; group 4). The fifth group contains a single ACDMPV case with no known *FOXF1* abnormalities. In particular, heterozygous CNV deletion on 16q24.1, involving* FOXF1* and its enhancer, was identified in patient 3.2. The deletion arose de novo on maternal chromosome 16. The genomic findings in the remaining ACDMPV neonates were described previously [16–18, 34–36]. Detailed sample description is presented in Additional file 1.

### *FENDRR* knockout in lung fibroblasts

*FENDRR* knockout in IMR-90 cells was performed using CRISPR/Cas9 system by deletion of a 0.6 kb region of chromosome 16q24.1 containing two thirds of the putative *FENDRR* promoter (candidate *cis*-Regulatory Element, cCRE, in ENCODE; http://www.encodeproject.org), the entire exon 1, and adjacent to it, a portion of the intron 1 (Additional file 3). Upstream and downstream single guide RNA (sgRNA) sequences (5’ TGTGAGTTCAGATCCGAGCG; chr16: 86,509,150–86,509,169, hg38 and 5’ GGTCATCGCGAAGAGCCTAG; chr16: 86,508,542–86,508,561, hg38, respectively), flanking the region to be deleted, were designed using the CRISPOR Design Tool (http://crispor.tefor.net/; [37]). Double stranded oligonucleotides (Integrated DNA Technologies, Coralville, IA), encoding guide RNAs, were cloned in the *Bbs*I sites of the pSpCas9(BB)-2A-GFP (pX458) plasmids (Addgene, Watertown, MA), which constitutively express Cas9 and GFP. A culture of 2 × 10^6^ IMR-90 lung fibroblasts was co-transfected with two pX458-derived vectors, expressing either upstream or downstream sgRNA, using Lipofectamine 3000 (Invitrogen, Carlsbad, CA). GFP-positive cells were collected 48 h later applying Fluorescence Assisted Cell Sorting (FACS) at the BCM Cytometry and Cell Sorting Core (on FACSAriaII sorter with nozzle size of 70 µm). The deletion was confirmed by PCR amplification and Sanger sequencing of the genomic region across the deletion junction. GFP-positive cells were subsequently processed to isolate total RNA for RNA-seq.

### RNA-seq based transcriptome profiling

The RNA-seq-based transcriptomic profiling was performed in 12 ACDMPV-affected lung samples and three age-matched normal lung controls (Additional file 1). Transcriptomic analyses were also performed in IMR-90 fetal lung fibroblasts with *FENDRR* knockout obtained using CRISPR/Cas9 system, and in IMR-90 cells in which *FENDRR* expression was reduced by applying RNA interference (RNAi) targeting spliced and un-spliced *FENDRR* transcripts [33]. RNA-seq experiments were performed using the Illumina NovaSeq 6000 System at BCM Genomic and RNA Profiling Core.

RNA-seq raw reads were mapped to the human genome (GRCh38) using STAR aligner (v 2.7.0). To generate the counts of reads uniquely mapped to the known and annotated genes, the featureCount method (R package Rsubread) and the Ensembl annotation file GRCh38.95.gtf were used. The count table of the uniquely mapped reads was generated and differential expression was tested using the DESeq2 (R Bioconductor package). Heatmaps were generated using the ClustVis online tool at https://biit.cs.ut.ee/clustvis/ [38]. To perform the Principal Component Analysis (PCA), the count data were transformed using the VarianceStabilizingTransformation method and visualized using the plotPCA method (both implemented in DESeq2 package).

### Pathway over-representation analysis across differentially expressed genes

The over-representation analysis of molecular pathways in each group was performed using the ConsensusPathDB server [39] accessible at http://ConsensusPathDB.org. Collection of annotated gene sets for further pathway assessment were obtained through the Molecular Signatures Database (MSigDB, v7.2, http://www.gsea-msigdb.org/gsea/msigdb/index.jsp) [40, 41]. Functional annotation of deregulated genes in Groups 1–5 was determined by Gene Ontology (GO) enrichment analyses using the clusterProfiler [42].

### Validation of RNA-Seq data by targeted gene expression analysis using NanoString

Verification of RNA-seq data was done using nCounter platform (NanoString Technologies, Seattle, WA). A hundred and twenty selected genes, whose expression was found by RNA-seq to be mis-regulated in ACDMPV patients, were included in the custom code sets. *GAPDH, ACTB*, and *PGK1* were used as the reference for gene expression normalization. To compare NanoString and RNA-seq data, the average signal obtained in NanoString experiment was separately calculated for three patients with ACDMPV (1.1, 4.1, and 4.2) and three controls (C1, C3, and C4). All RNA samples used for NanoString experiment have been assessed also in the prior RNA-Seq studies. The ratio between the average signal from NanoString has been compared to the ratio of the average normalized RNA-seq read count in patients and controls.

### Comparison of RNA-seq and microarray data

To compare the results of this RNA-seq and of our previous expression microarrays analysis [27], we have selected the genes that were consistently either up-regulated or down-regulated in both datasets. We have compared protein coding genes deregulated in each ACDMPV group (adjusted p-value < 0.01; log2 fold change > 1.0 or < − 1.0) with the genes deregulated in ACDMPV patients in the microarray study (adjusted p-value < 0.01). For the RNA-seq data, we have required that both direction of deregulation are consistent among all five ACDMPV groups.

## Results

### Perturbations of ACDMPV transcriptomes

**Fig. 1:**
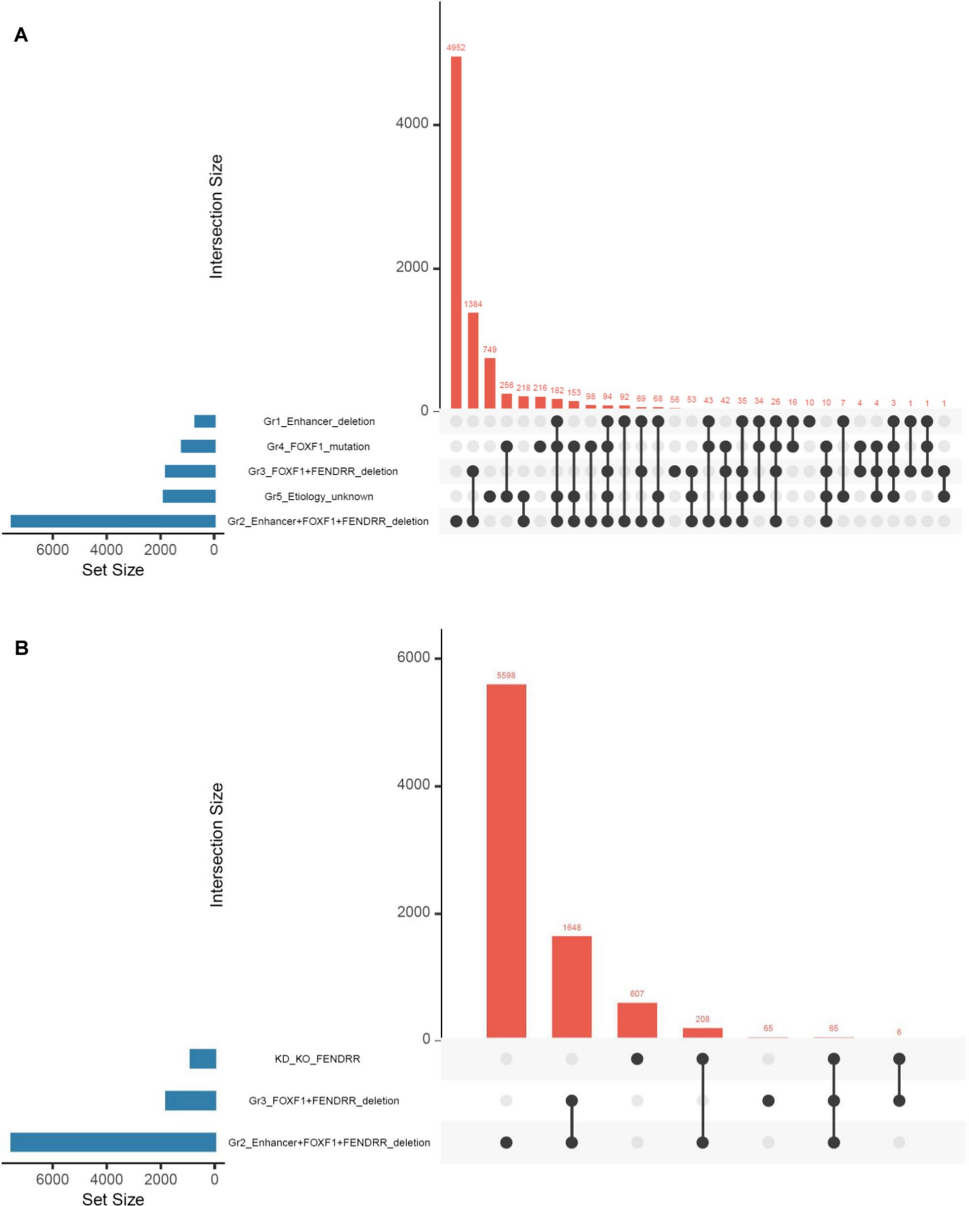
Upset plots showing intersections of differentially expressed genes between **A** each ACDMPV group and **B** IMR-90 cells with *FENDRR* knockdown (KD) and knockout (KO) and lung tissues of patients with ACDMPV and *FENDRR* deletion (Groups 2 and 5)

The number of reads that were uniquely mapped to the reference human genome varied between 5.6 and 40.1 million per sample in Group 1, 6.5 and 13.8 million in Group 2, 4.8 and 16.8 million in Group 3, 30.9 and 36.2 million in Group 4, and were 120.5 million in Group 5. Statistical analyses of RNA-seq data revealed on average ~ 2,600 differentially expressed genes in each ACDMPV group compared to normal control samples (adjusted p-value < 0.01; log2 fold change > 1.0 or < − 1.0). The highest number of deregulated protein-coding genes was observed in group 2 (n = 7525), while the lowest number was detected in group 1 (n = 682) (Additional file 4). The visual summary of the differentially expressed genes in each group is presented in Fig. 1A.

**Fig. 2:**
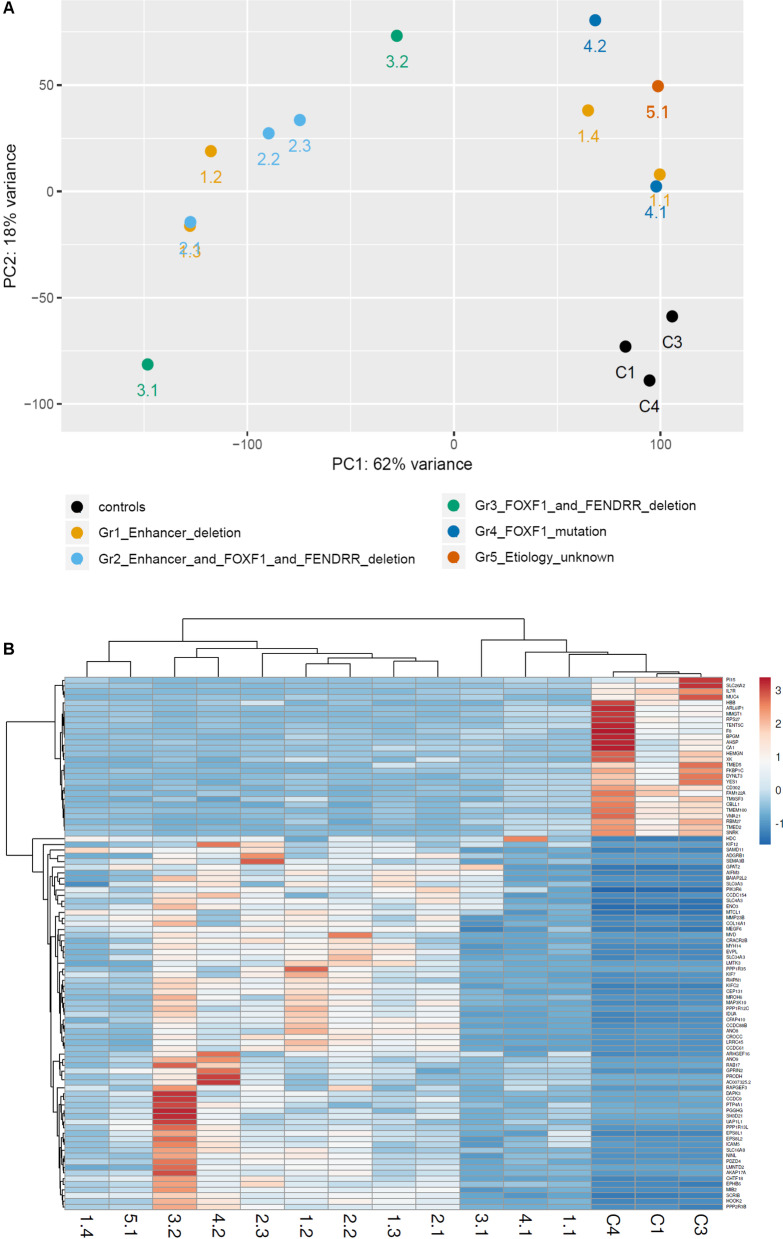
Results of comparative gene expression analyses. **A** Principal Component Analysis (PCA) of RNA-seq data in lungs from 12 ACDMPV patients. Pt 3.1 is an outlier (low QC score); **B** Heatmap indicating hierarchical clustering of ACDMPV and non-ACDMPV control samples based on the RNA-seq expression values of common 94 genes showing deregulation in each ACDMPV sample

PCA showed that all analyzed ACDMPV cases clustered away from the normal lung controls (Fig. 2A). While group 2 (CNV deletion of *FOXF1* and its enhancer) and group 4 (*FOXF1* mutations) form separate clusters, group 1 (CNV deletion involving *FOXF1* enhancer) and group 3 (CNV deletion involving *FOXF1*) are scattered (Fig. 2A).

Ninety-four genes were commonly deregulated in all ACDMPV individuals (Additional file 5). Notably, a changed expression of genes involved in vascular patterning or epithelial branching, *TMEM100*, *SEMA3B*, and *EPHB6*, was found in each patient. Deregulation of several genes previously described as involved in lung extracellular matrix formation (*COL16A1* and *MEGF6*), lung cancer (*IL7R* and *YES1*), pulmonary fibrosis (*MMP23B*, *PTP4A1*, and *MUC4*), or chronic obstructive pulmonary disease (*LRRC45* and *MTCL1*) was also observed in each group (Fig. 2B).

### Pathway over-representation analysis across differentially expressed genes in ACDMPV patients

Over-representation analyses performed on the combined lists of upregulated and downregulated transcripts revealed significant enrichment of differentially expressed protein-coding genes from several molecular pathways linked to lung development and function (Additional file 6).

In group 1, a significant over-representation includes the genes encoding axon guidance proteins—ephrins (EPHs), semaphorins (SEMAs), and plexins (PLXNs), hedgehog signaling pathway—*SHH* and *KIF7*, and proteins involved in O_2_/CO_2_ transport—*HBA1*, *CA1*, and *HBB*, whereas in group 2 those involved in TGF-beta and WNT signaling (*TGFB1*, *TGFBR1-3*, *BMPR2*, *CTNNB1*, *ACVR2A*, *ACVR2B*, *SMAD*, and *MAPK* genes, *FGF6* and *ANGPTL1*), PDGF and vascular endothelial growth factor (VEGF) signaling (*PDGFC*, *VEGFC*, and *VEGFD*), SEMA-neuropilin (SEMA-NRP) signaling (*SEMA3B* and *NRP1*) as well as genes involved in NOTCH (*NOTCH2*) and SHH (*SHH*) signaling were detected. In group 3, we have found deregulated genes that encode proteins involved in NOTCH3 signaling (*APH1A* and *SNW1*), TGF-beta, and SMAD, and MAPK signaling (*TGFBR1*, *SP1*, and *MAPK1*) and in group 4 those, involved in axon guidance and SEMA and PLXN receptors, and IL-17 signaling pathway (MAPK and IL17 receptors). Among pathways affected in group 5, there were axon guidance and SEMA signaling (*PLXN* genes), SHH and NOTCH signaling (*GLI2*, *KIF7,* and *NOTCH3*), ephrin signaling (*EPHA2*, *EPHA1*, *EPHB3*, and *YES1*), VEGF signaling (*MMP14*, *HDAC5*, and *JUN*), and MAPK signaling (*MAP2K2* and *TLN1*) (Additional file 6).

Differentially expressed genes were subjected to the GO enrichment analyses. We have found 20 enriched GO terms for Group 1, 619 for Group 2, 254 for Group 3, 82 for Group 4, and 282 for Group 5. The top overrepresented GO biological processes (BP) include regulation of GTPase activity (Groups 1 and 4), mRNA catabolic process (Group 2), translational initiation (Group 3), and extracellular structure organization (Group 5) (Additional file 7). Among other significant GO BP terms was semaphorin–plexin signaling pathway involved in axon guidance (Group 4). Within GO molecular function (MF), the most enriched ones were associated with small GTPase binding (Groups 1 and 4), cadherin binding (Group 2), structural constituent of ribosome (Group 3), and actin binding (Group5) (Additional file 7).

Hierarchical clustering of genes from commonly deregulated pathways: axon guidance (R-HSA-422475), SEMA interactions (R-HSA-373755), and VEGF signaling (R-HSA-194138) showed separation of ACDMPV and non-ACDMPV samples (Additional file 8, Additional file 9, Additional file 10).

### Deregulation of genes in *FENDRR*-depleted lung fibroblasts

Analysis of the RNA-seq data for IMR-90 cells with *FENDRR* knockdown and knockout [33] revealed deregulation of 886 protein-coding genes with log2 fold change > 1.0 or < − 1.0. These genes were significantly over-represented in several molecular pathways, including signaling by NOTCH2 (*HES5* and *FCER2*) or JAK-STAT signaling (*CSF2RA, IL10*, *IL13*, and *IL23R*) (Additional file 11). Comparison of gene expression patterns in *FENDRR*-depleted IMR-90 cells (knockout and knockdown) and lung tissues (ACDMPV patients with *FENDRR* deletion; Groups 2 and 3) revealed 65 commonly deregulated genes (Fig. 1B).

### Validation of the RNA-Seq results by NanoString assay

The 120 values for analyzed genes obtained by both methods showed a strong correlation between the two sets of results (Additional file 12).

### Comparison of RNA-seq data with microarray-based expression results

We have found a partial overlap between the RNA-seq results and our previous expression microarray data [27]. In Group 1 we have identified 46 genes that were commonly deregulated in both datasets. In Groups 2, 3, 4, and 5, we have detected 379, 77, 76, and 144 differentially expressed genes, respectively, that were also found deregulated in the microarray study. (Additional file 13).

## Discussion

In this study, we have performed a comprehensive RNA-seq profiling in the lung samples from 12 ACDMPV patients, classified into five separate categories based on the type of the *FOXF1* defect. While we have identified the differences between the transcriptomic profiles in ACDMPV lungs when compared to the control tissues, a substantial difference in gene expression levels among ACDMPV study groups was also observed. Nonetheless, 94 genes were significantly deregulated in all ACDMPV individuals, suggesting their contribution to the disease. Those genes can be used as a reference set in the diagnosis of ACDMPV cases in which neither causative SNV nor CNV deletion involving the *FOXF1* locus is detected.

In all ACDMPV lung specimens, we have found an increased expression of *SEMA3B*. SEMA3B belongs to the semaphorin family which, besides being involved in axon guidance signaling, was also found to be responsible for murine lung bud formation and normal alveolar development [43]. While the role of SEMA3B in lung growth is not well characterized, SEMA3A was found to inhibit branching morphogenesis of fetal mouse lung [44] whereas SEMA3C and SEMA3F were reported to promote airway branching and increase proliferation of terminal epithelial cells [45–47]. Of note, in this study, *SEMA3C* and other SEMA genes were also significantly deregulated in ACDMPV patients with deletion of *FOXF1* and its distant lung-specific enhancer.

Semaphorins act mostly through binding with PLXNs and NRPs, their primary receptors and co-receptors, respectively [48, 49]. Notably, in all but one studied ACDMPV groups, PLXNs, including *PLXNA3*, *PLXNB1-3*, and *PLXND1*, were found to be significantly deregulated. Also, the expression level of the *NRP1* receptor, recently shown to facilitate SARS-CoV-2 cell entry [50], was decreased in groups 2 and 3. In support of this notion, disruption of SEMA-NRP1 signaling in murine lungs was reported to cause instability of the alveolar-capillary interface and hypertensive remodeling reminiscent of ACD [51, 52]. Our data on SEMA-NRP1 deregulation further imply the role of this signaling pathway in lung development.

Deregulation of *SEMA*s, *PLXN*s, and *NRP1* in ACDMPV patients with various *FOXF1* abnormalities is supported by our previous ChIP-seq analyses performed in E18.5 lungs of mice overexpressing *Foxf1* that revealed direct interactions of FOXF1 with their loci [53]. In addition, in two unrelated ACDMPV neonates without any detectable variant in *FOXF1*, we previously reported the c.631C > G (p.Leu211Val) and c.3256G > A (p.Ala1086Thr) variants in *PLXNB2*, inherited from their healthy fathers [16]. Overall, these data imply potential interaction between the SHH-FOXF1 and SEMA-NRP signaling pathways.

In addition to SEMAs, also VEGFs are ligands to PLXNs, and NRP1. VEGF controls pulmonary vasculogenesis and its inhibition in rats resulted in alveolar simplification and loss of lung capillaries [54]. Decreased expression of the VEGF receptor gene, *Flt1*, was found in the *Foxf1* conditional knockout mouse endothelium, implying that FOXF1 can regulate endothelial genes involved in VEGF signaling [55]. Here, downregulation of *FLT1* was observed in ACDMPV patients with the *FOXF1* enhancer deletions, further suggesting that this gene can be regulated by the FOXF1 pathway. Of note, VEGF signaling is connected to the axon guidance pathway through NRPs, which can also bind members of the VEGF family [56].

*EPHB6*, a member of the ephrin family that is upregulated in all ACDMPV patients, similarly to SEMAs, is also involved in axon guidance signaling. A possible role of EPHs as molecular stimulators of lung morphogenesis was reported in rat lungs [57]. In humans, *EPHB6* is implicated in inhibition of metastasis in several types of cancer, including non-small cell lung cancer [58].

In all ACDMPV newborns, we have found a decreased level of lung-specific *TMEM100* encoding Transmembrane protein 100. In humans and mice, this gene was found to be most abundantly expressed in the lungs (Additional file 14) [59]. It was demonstrated that TMEM100 acts downstream of BMP9/BMP10-ALK1 (ACVRL1) signaling pathway, necessary for murine embryonic arterial endothelium differentiation, vascular development, and early lung morphogenesis [60, 61]. Interestingly, mutations in genes in the BMP pathway are associated with PAH [62], which is often part of the clinical manifestation of ACDMPV. Importantly, *TMEM100* is also a potential downstream target of FOXF1 detected by ChIP-seq in mice overexpressing *Foxf1* [53], suggesting that *FOXF1* abnormalities may trigger expression changes of this gene in ACDMPV patients.

Among the most prominent pathways with the over-represented genes detected in one or more studied ACDMPV groups is SHH signaling, one of the key pathways regulating lung formation [28]. During mouse pulmonary morphogenesis, molecular signaling from epithelial SHH to the mesenchymal *FOXF1* is critical for normal pulmonary vasculature development [23, 63]. The increase of SHH pathway expression in ACDMPV could represent a compensatory response to the loss of FOXF1. In addition to *FOXF1*, SHH is thought to control expression of the *TBX* genes, which are also associated with LLDD and PAH in humans [64–69] and are known regulators of lung branching in mice [70, 71]. Of note, in our recent ChIP-seq study performed in IMR-90 fetal lung fibroblasts, we demonstrated that TBX2 and/or TBX4 bind to *FOXF1*, *FENDRR*, their promoter(s) and lung-specific enhancer, as well as to genes from the axon guidance/SEMA signaling *(SEMA*s, *PLXN*s, and *NRP*), that were found to be deregulated in ACDMPV patients in this study. This suggests that SHH-FOXF1, SEMA-NRP, and the TBX2-TBX4 signaling may be involved in a crosstalk responsible for normal human lung development, disruption of which leads to LLDD.

We have found also a decrease of WNT signaling, especially the canonical WNT signaling to β-catenin, one of the pathways mediating crosstalk between lung epithelia and mesenchyme during branching and important for pulmonary angiogenesis [72].

Noteworthy is also a decrease of *S1PR1* expression. *S1PR1* is essential for blood/air barrier functioning and its murine orthologue was found downregulated in the *Foxf1*^+/-^ mice [73]. The detected increase of* NKX2-1* expression might account for both lung and cardiac abnormalities in ACDMPV patients. *NKX2-1* has been associated with severe abnormalities in early development of lungs [74] and found mutated in patients with cardiac septum defects [75]. *NKX2-5*, which has been associated with hypoplastic left heart syndrome [76], was upregulated in ACDMPV patients with deletion of *FOXF1* and its enhancer.

*FENDRR* shares with *FOXF1* the distant lung-specific enhancer and promoter-containing region and its transcription was found to be positively regulated by FOXF1 [33]. To better characterize the role of *FENDRR* in lung development, we have analyzed transcriptome perturbations in the IMR-90 fetal lung fibroblast cells depleted of *FENDRR* using siRNA or CRISPR/Cas9 since we did not have a patient-derived material with an isolated *FENDRR* deletion. We have chosen IMR-90 cells because of their relatively high expression of *FENDRR* and *FOXF1* and their origin from normal fetal lungs in pseudoglandular and canalicular stages of lung development at which ACDMPV abnormalities are first observed. One of the upregulated genes in cells with knockdown and knockout of *FENDRR* is *HES5*, a downstream target of Notch signaling [77]. High level of the HES5 protein was observed in lungs of patients with PAH. Another gene deregulated in lung fibroblasts with the reduced level of *FENDRR* is *CSF2RA* that was previously involved in pulmonary alveolar proteinosis [78]. Comparison between fibroblasts with *FENDRR* knockout and knockdown and lung tissues from ACDMPV patients with *FENDRR* deletion revealed a common dysregulation of 65 genes. Nearly 50% of these genes have the same direction of changes in both groups whereas the other half of genes were deregulated in the opposite direction. That could be caused by a specific expression profiles among different cell types.

Interestingly, many gene expression changes identified in lung transcriptome from the ACDMPV patient with normal levels of *FOXF1* and *FENDRR* transcripts and without any pathogenic variant involving *FOXF1*, *FENDRR*, or their distant enhancer (Group 5) overlapped with those found in ACDMPV cases related to *FOXF1* deficiency, suggesting a defect involving the FOXF1 target in patient 5.1.

Here, we have expanded our previously reported targeted expression array-based ACDMPV study [27] by global transcriptome RNA-seq profiling. A partial overlap between both datasets was observed. However, since we have used fewer samples in our previous experiment than in the current RNA-seq analysis, the differences in gene expression levels in RNA-seq and microarray are not unexpected. Overall, RNA-seq provided more detailed information on specific transcript expression patterns, confirmed by a validation experiment using NanoString, and showed a significant correlation between both methods.

This is the first study of ACDMPV patients providing the comprehensive transcriptome profiles of samples with various genetic variants involving *FOXF1* and/or its regulatory region. However, our analyses are based on a limited number of samples and further studies involving more patients should be performed to draw more robust conclusions. While the observed differences in transcriptome patterns of patients may be partially explained by variability in type and/or size of genomic defects within the 16q24.1 locus, the gene expression profiles may be affected by the discrepancies in the number of sequencing reads in each sample due to the quality differences of obtained tissues. The transcriptome profiles of the samples with a low number of reads (< 10 M) are less accurate when compared to those with a higher read depth and their inclusion could reduce the statistical power by increasing the number of false negatives. Nevertheless, we expect that a low number of reads in the selected samples should not impact the false positive rate that may be much better controlled by a strict algorithm applied for the selection of genes with differential expression.

## Conclusions

Our transcriptomic analyses provide further insight into the complexity of the network of signaling pathways affected by *FOXF1* deficiency in ACDMPV patients. Overall, consistent with the absence of major phenotypic differences among patients with CNV deletions and point mutations in *FOXF1* [16], our RNA-seq analyses further indicate that *FOXF1* deficiency is pathogenic for ACDMPV. The partial overlap of transcriptome changes between ACDMPV lungs and *FENDRR*-depleted IMR-90 cells suggests the involvement of this lncRNA in the ACDMPV etiology.

## Supplementary Information

**Additional file 1.** ACDMPV patients enrolled in transcriptomic studies.

**Additional file 2.** Schematic representation of CNVs and point mutations detected within *FOXF1* locus in ACDMPV patients enrolled in transcriptomic studies.

**Additional file 3.** CRISPR/Cas9 knockout of *FENDRR* in IMR-90 cell line in IMR-90 cell line. **A** Schematic representation of the *FENDRR* locus and CRISPR/Cas9-mediated *FENDRR* knockout strategy. Red box represents *FENDRR* exon 1; white box represents maximal 5’ extension of the exon 1, caused by alternative splicing, that is present in GENCODE transcript *FENDRR-206* (ENST00000599749.5). F and R indicate primers (F: 5’-GAAAATGAAGGCGTCCGAGC, R: 5’-GCACATGCAAACCCGGTAAA) used in PCR amplification from normal (WT) and edited (KO) lung fibroblasts IMR-90. **B** Agarose gel electrophoresis of PCR products that verified promoter-exon 1 deletion (pe1∆). There was a minor heterogeneity with respect to the exact position of each of the deletion breakpoints. Sequence through the deletion junction and deletion coordinates refer to the predominant deletion (a small fraction of the deletions was 2 to 4 nt shorter). cCRE abbreviates *FENDRR* putative promoter (ENCODE).

**Additional file 4.** List of protein coding genes deregulated in ACDMPV lungs. Case grouping is described in Additional file 1.

**Additional file 5.** List of 94 genes commonly deregulated in all ACDMPV individuals.

**Additional file 6.** Molecular pathways affected in ACDMPV lungs, identified by RNA-seq (ConsensusPathDB analysis). Case grouping is described in Additional file 1.

**Additional file 7.** Gene ontology analysis of differentially expressed genes in each group. A bar chart of the most significant gene ontology (GO) biological processes (left panel) and molecular function (right panel) terms for differentially expressed genes.

**Additional file 8.** Heatmap indicating hierarchical clustering of ACDMPV and non-ACDMPV control samples based on the RNA-seq expression values of genes from axon guidance (R-HSA-422475).

**Additional file 9.** Heatmap indicating hierarchical clustering of ACDMPV and non-ACDMPV control samples based on the RNA-seq expression values of genes from semaphorin interaction (R-HSA-373755).

**Additional file 10.** Heatmap indicating hierarchical clustering of ACDMPV and non-ACDMPV control samples based on the RNA-seq expression values of genes from VEGF signaling (R-HSA-194138).

**Additional file 11.** Molecular pathways affected in IMR-90 cells with* FENDRR* depletion, identified by RNA-seq (ConsensusPathDB analysis).

**Additional file 12.** Mutual comparison of RNA-seq and NanoString nCounter-determined changes in ACDMPV transcriptomes. Both methods detected relatively similar changes in expression levels of selected 120 genes.

**Additional file 13.** Comparison of RNA-seq data with microarray-based expression results (GSE54780). Case grouping is described in Additional file 1.

**Additional file 14.** Bio-GPS graphs demonstrating similar fetal lung- and lung-specific expression pattern of *TMEM100* and *FOXF1*.

## Data Availability

The datasets used and/or analyzed during the current study are available from the corresponding author on reasonable request.

## Abbreviations

ACDMPV: Alveolar capillary dysplasia with misalignment of pulmonary veins
Array CGH: Array comparative genomic hybridization
BCM: Baylor College of Medicine
CNV: Copy-number variant
EPH: Ephrin
FOX: Forkhead-box family transcription factor
FFPE: Formalin-fixed paraffin-embedded
lncRNA: Long noncoding RNA
LLDD: Lethal lung developmental disorder
NRP: Neuropilin
PAH: Pulmonary arterial hypertension
PLXN: Plexin
RNA-seq: RNA sequencing
RNAi: RNA interference
SEMA: Semaphorin
sgRNA: Single guide RNA
SHH: Sonic hedgehog
SNV: Single nucleotide variant
SNP: Single nucleotide polymorphism
VEGF: Vascular endothelial growth factor
