# Supplementary material for: Perturbation of semaphorin and VEGF signaling in ACDMPV lungs due to FOXF1 deficiency
Source: Respir Res. 2021 Jul 27;22:212. doi: 10.1186/s12931-021-01797-7 (PMC8314029; doi:10.1186/s12931-021-01797-7)
Supplement: Supplementary file 1 — Additional file 1. ACDMPV patients enrolled in transcriptomic studies. [file 12931_2021_1797_MOESM1_ESM.pdf]

Additional file 1. ACDMPV patients enrolled in transcriptomic studies.

**Patient / Sample**	**Lab Number**	**Group (Molecular defect at chr16q24.1)**	**Coordinates (hg38)**	**Parental origin of the chromosome 16, carrying pathogenic variant**	**Reference**
C1	C1	Controls	N/A	N/A	N/A
C3	C3		N/A	N/A	N/A
C4	C4		N/A	N/A	N/A
1.1	60.4	1 (Enhancer deletion)	chr16:83,639,777/871-86,264,678/772	Maternal	Szafranski et al. 2013
1.2	28.7		~chr16:86,106,893-86,251,893	Maternal	Stankiewicz et al. 2009
1.3	155.3		chr16:84,457,588/632-86,238,392/436	Maternal	Szafranski et al. 2018
1.4	179.3		chr16:83,637,918/969-86,262,821/872	Paternal	Szafranski et al. 2018
2.1	90.3	2 (Enhancer + *FOXF1* + *FENDRR* deletion)	chr16:81,061,049-86,603,147	Maternal	Sen et al. 2013
2.2	118.3		chr16:85,993,409/427-87,165,121/139	Maternal	Prothro et al. 2016
2.3	170.3		chr16:85,030,576-86,687,561	Maternal	Szafranski et al. 2018
3.1	115.3	3 (*FOXF1* + *FENDRR* deletion)	chr16:86,471,844-86,541,855	Maternal	Szafranski et al. 2016
3.2	176.3		~chr16:86,508,525-86,515,660	Maternal	This work
4.1	73.3	4 (*FOXF1* mutation)	NM_001451.3:c.302C>T p.Ser101Leu	-	Sen et al. 2013
4.2	123.3		NM_001451.3:c.849_850del p.Ile285Glnfs*9	-	Szafranski et al. 2016
5.1	20.8	5 (Etiology unknown)	N/A	N/A	This work

N/A, not applicable; -, data not available.

Additional References:

Prothro SL, Plosa E, Markham M, Szafranski P, Stankiewicz P, Killen SAS. 2016. Prenatal Diagnosis of Alveolar Capillary Dysplasia with Misalignment of Pulmonary Veins. *The Journal of Pediatrics* 170:317–318. DOI: 10.1016/j.jpeds.2015.11.041.

Sen P, Yang Y, Navarro C, Silva I, Szafranski P, Kolodziejska KE, Dharmadhikari AV, Mostafa H, Kozakewich H, Kearney D, Cahill JB, Whitt M, Bilic M, Margraf L, Charles A, Goldblatt J, Gibson K, Lantz PE, Garvin AJ, Petty J, Kiblawi Z, Zuppan C, McConkie-Rosell A, McDonald MT, Peterson-Carmichael SL, Gaede JT, Shivanna B, Schady D, Friedlich PS, Hays SR, Palafoll IV, Siebers-Renelt U, Bohring A, Finn LS, Siebert JR, Galambos C, Nguyen L, Riley M, Chassaing N, Vigouroux A, Rocha G, Fernandes S, Brumbaugh J, Roberts K, Ho-Ming L, Lo IFM, Lam S, Gerychova R, Jezova M, Valaskova I, Fellmann F, Afshar K, Giannoni E,

Muhlethaler V, Liang J, Beckmann JS, Lioy J, Deshmukh H, Srinivasan L, Swarr DT, Sloman M, Shaw-Smith C, van Loon RL, Hagman C, Sznajer Y, Barrea C, Galant C, Detaille T, Wambach JA, Cole FS, Hamvas A, Prince LS, Diderich KEM, Brooks AS, Verdijk RM, Ravindranathan H, Sugo E, Mowat D, Baker ML, Langston C, Welty S, Stankiewicz P. 2013. Novel *FOXF1* mutations in sporadic and familial cases of alveolar capillary dysplasia with misaligned pulmonary veins imply a role for its DNA binding domain. *Human Mutation* 34:801–811. DOI: 10.1002/humu.22313.

Stankiewicz P, Sen P, Bhatt SS, Storer M, Xia Z, Bejjani BA, Ou Z, Wiszniewska J, Driscoll DJ, Maisenbacher MK, Bolivar J, Bauer M, Zackai EH, McDonald-McGinn D, Nowaczyk MMJ, Murray M, Hustead V, Mascotti K, Schultz R, Hallam L, McRae D, Nicholson AG, Newbury R, Durham-O'Donnell J, Knight G, Kini U, Shaikh TH, Martin V, Tyreman M, Simonic I, Willatt L, Paterson J, Mehta S, Rajan D, Fitzgerald T, Gribble S, Prigmore E, Patel A, Shaffer LG, Carter NP, Cheung SW, Langston C, Shaw-Smith C. 2009. Genomic and genic deletions of the *FOX* gene cluster on 16q24.1 and inactivating mutations of *FOXF1* cause alveolar capillary dysplasia and other malformations. *American Journal of Human Genetics* 84:780–791. DOI: 10.1016/j.ajhg.2009.05.005.

Szafranski P, Dharmadhikari AV, Brosens E, Gurha P, Kolodziejska KE, Zhishuo O, Dittwald P, Majewski T, Mohan KN, Chen B, Person RE, Tibboel D, de Klein A, Pinner J, Chopra M, Malcolm G, Peters G, Arbuckle S, Guiang SF, Hustead VA, Jessurun J, Hirsch R, Witte DP, Maystadt I, Sebire N, Fisher R, Langston C, Sen P, Stankiewicz P. 2013. Small noncoding differentially methylated copy-number variants, including lncRNA genes, cause a lethal lung developmental disorder. *Genome Research* 23:23–33. DOI: 10.1101/gr.141887.112.

Szafranski P, Gambin T, Dharmadhikari AV, Akdemir KC, Jhangiani SN, Schuette J, Godiwala N, Yatsenko SA, Sebastian J, Madan-Khetarpal S, Surti U, Abellar RG, Bateman DA, Wilson AL, Markham MH, Slamon J, Santos-Simarro F, Palomares M, Nevado J, Lapunzina P, Chung BH-Y, Wong W-L, Chu YWY, Mok GTK, Kerem E, Reiter J, Ambalavanan N, Anderson SA, Kelly DR, Shieh J, Rosenthal TC, Scheible K, Steiner L, Iqbal MA, McKinnon ML, Hamilton SJ, Schlade-Bartusiak K, English D, Hendson G, Roeder ER, DeNapoli TS, Littlejohn RO, Wolff DJ, Wagner CL, Yeung A, Francis D, Fiorino EK, Edelman M, Fox J, Hayes DA, Janssens S, De Baere E, Menten B, Loccufier A, Vanwalleghem L, Moerman P, Sznajer Y, Lay AS, Kussmann JL, Chawla J, Payton DJ, Phillips GE, Brosens E, Tibboel D, de Klein A, Maystadt I, Fisher R, Sebire N, Male A, Chopra M, Pinner J, Malcolm G, Peters G, Arbuckle S, Lees M, Mead Z, Quarrell O, Sayers R, Owens M, Shaw-Smith C, Lioy J, McKay E, de Leeuw N, Feenstra I, Spruijt L, Elmslie F, Thiruchelvam T, Bacino CA, Langston C, Lupski JR, Sen P, Popek E, Stankiewicz P. 2016. Pathogenetics of alveolar capillary dysplasia with misalignment of pulmonary veins. *Human Genetics* 135:569–586. DOI: 10.1007/s00439-016-1655-9.

Szafranski P, Kośmider E, Liu Q, Karolak JA, Currie L, Parkash S, Kahler SG, Roeder E, Littlejohn RO, DeNapoli TS, Shardonofsky FR, Henderson C, Powers G, Poisson V, Bérubé D, Oligny L, Michaud JL, Janssens S, De Coen K, Van Dorpe J, Dheedene A, Harting MT, Weaver MD, Khan AM, Tatevian N, Wambach J, Gibbs KA, Popek E, Gambin A, Stankiewicz P. 2018. LINE- and Alu-containing genomic instability hotspot at 16q24.1 associated with recurrent and nonrecurrent CNV deletions causative for ACDMPV. *Human Mutation* 39:1916–1925. DOI: 10.1002/humu.23608.
