# Supplementary material for: Perturbation of semaphorin and VEGF signaling in ACDMPV lungs due to FOXF1 deficiency
Source: Respir Res. 2021 Jul 27;22:212. doi: 10.1186/s12931-021-01797-7 (PMC8314029; doi:10.1186/s12931-021-01797-7)
Supplement: Supplementary file 2 — Additional file 2. Schematic representation of CNVs and point mutations detected within FOXF1 locus in ACDMPV patients enrolled in transcriptomic studies. [file 12931_2021_1797_MOESM2_ESM.pdf]

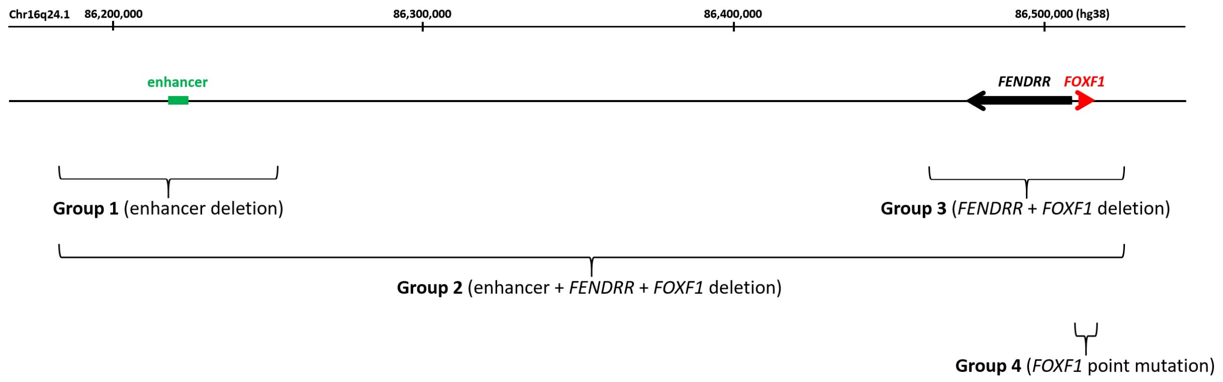

Additional file 2. Schematic representation of CNVs and point mutations detected within *FOXF1* locus in ACDMPV patients
