# Supplementary material for: Perturbation of semaphorin and VEGF signaling in ACDMPV lungs due to FOXF1 deficiency
Source: Respir Res. 2021 Jul 27;22:212. doi: 10.1186/s12931-021-01797-7 (PMC8314029; doi:10.1186/s12931-021-01797-7)
Supplement: Supplementary file 7 — Additional file 7. Gene ontology analysis of differentially expressed genes in each group. A bar chart of the most significant gene ontology (GO) biological processes (left panel) and molecular function (right panel) terms for differentially expressed genes. [file 12931_2021_1797_MOESM7_ESM.pdf]

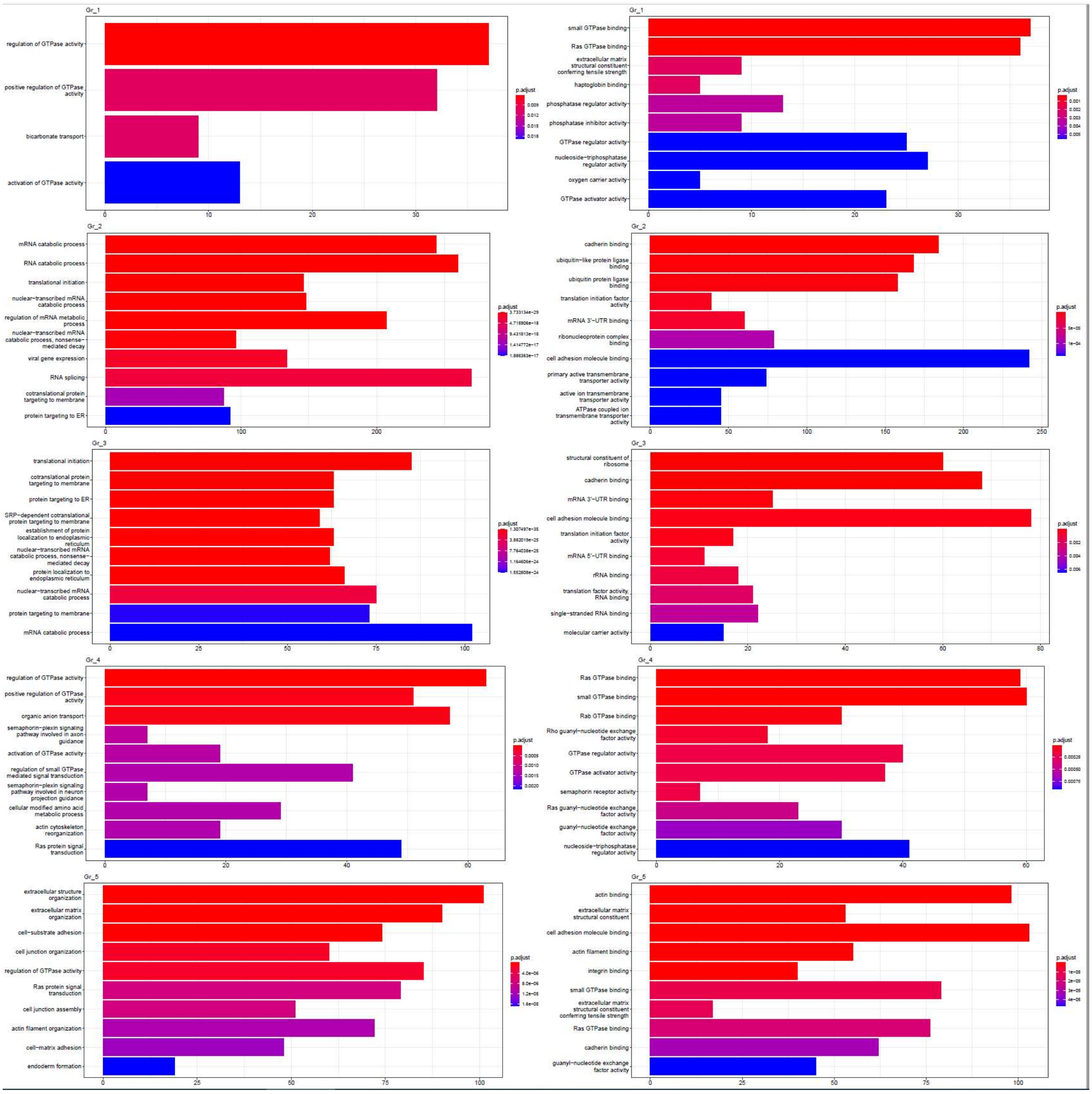


**Additional file 7.** Gene ontology analysis of differentially expressed genes in each group.
A bar chart of the most significant gene ontology (GO) biological processes (left panel) and molecular function (right panel) terms for differentially expressed genes.
