# Supplementary material for: Perturbation of semaphorin and VEGF signaling in ACDMPV lungs due to FOXF1 deficiency
Source: Respir Res. 2021 Jul 27;22:212. doi: 10.1186/s12931-021-01797-7 (PMC8314029; doi:10.1186/s12931-021-01797-7)
Supplement: Supplementary file 8 — Additional file 8. Heatmap indicating hierarchical clustering of ACDMPV and non-ACDMPV control samples based on the RNA-seq expression values of genes from axon guidance (R-HSA-422475). [file 12931_2021_1797_MOESM8_ESM.pdf]

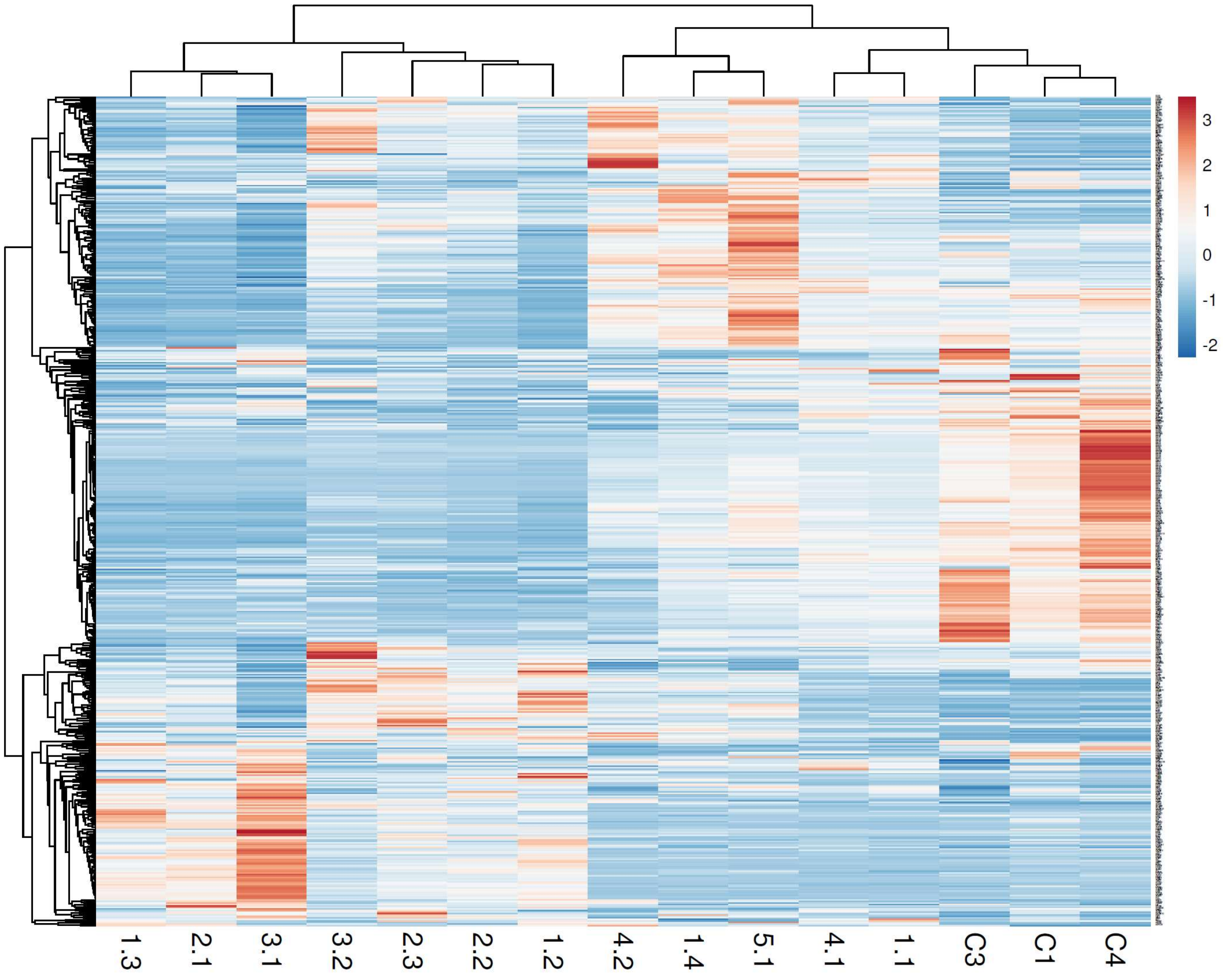

Additional file 8. Heatmap indicating hierarchical clustering of ACDMPV and non-ACDMPV control samples based on the RNA-seq expression values of genes from axon guidance (R-HSA-422475).
