# Supplementary material for: Perturbation of semaphorin and VEGF signaling in ACDMPV lungs due to FOXF1 deficiency
Source: Respir Res. 2021 Jul 27;22:212. doi: 10.1186/s12931-021-01797-7 (PMC8314029; doi:10.1186/s12931-021-01797-7)
Supplement: Supplementary file 9 — Additional file 9. Heatmap indicating hierarchical clustering of ACDMPV and non-ACDMPV control samples based on the RNA-seq expression values of genes from semaphorin interaction (R-HSA-373755). [file 12931_2021_1797_MOESM9_ESM.pdf]

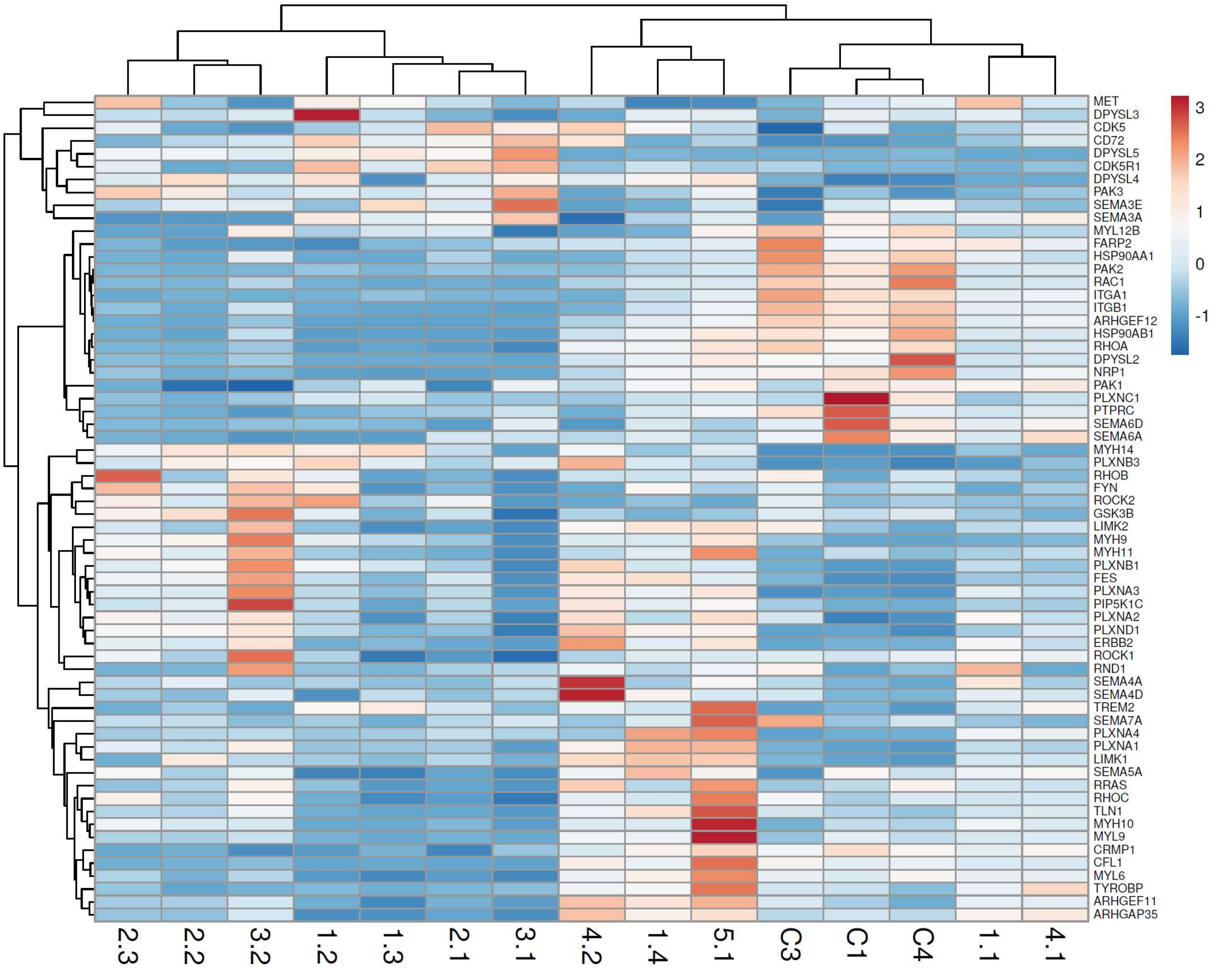

Additional file 9. Heatmap indicating hierarchical clustering of ACDMPV and non-ACDMPV control samples based on the RNA-seq expression values of genes from semaphorin interaction (R-HSA-373755).
