# Supplementary material for: Perturbation of semaphorin and VEGF signaling in ACDMPV lungs due to FOXF1 deficiency
Source: Respir Res. 2021 Jul 27;22:212. doi: 10.1186/s12931-021-01797-7 (PMC8314029; doi:10.1186/s12931-021-01797-7)
Supplement: Supplementary file 12 — Additional file 12. Mutual comparison of RNA-seq and NanoString nCounter-determined changes in ACDMPV transcriptomes. Both methods detected relatively similar changes in expression levels of selected 120 genes. [file 12931_2021_1797_MOESM12_ESM.pdf]

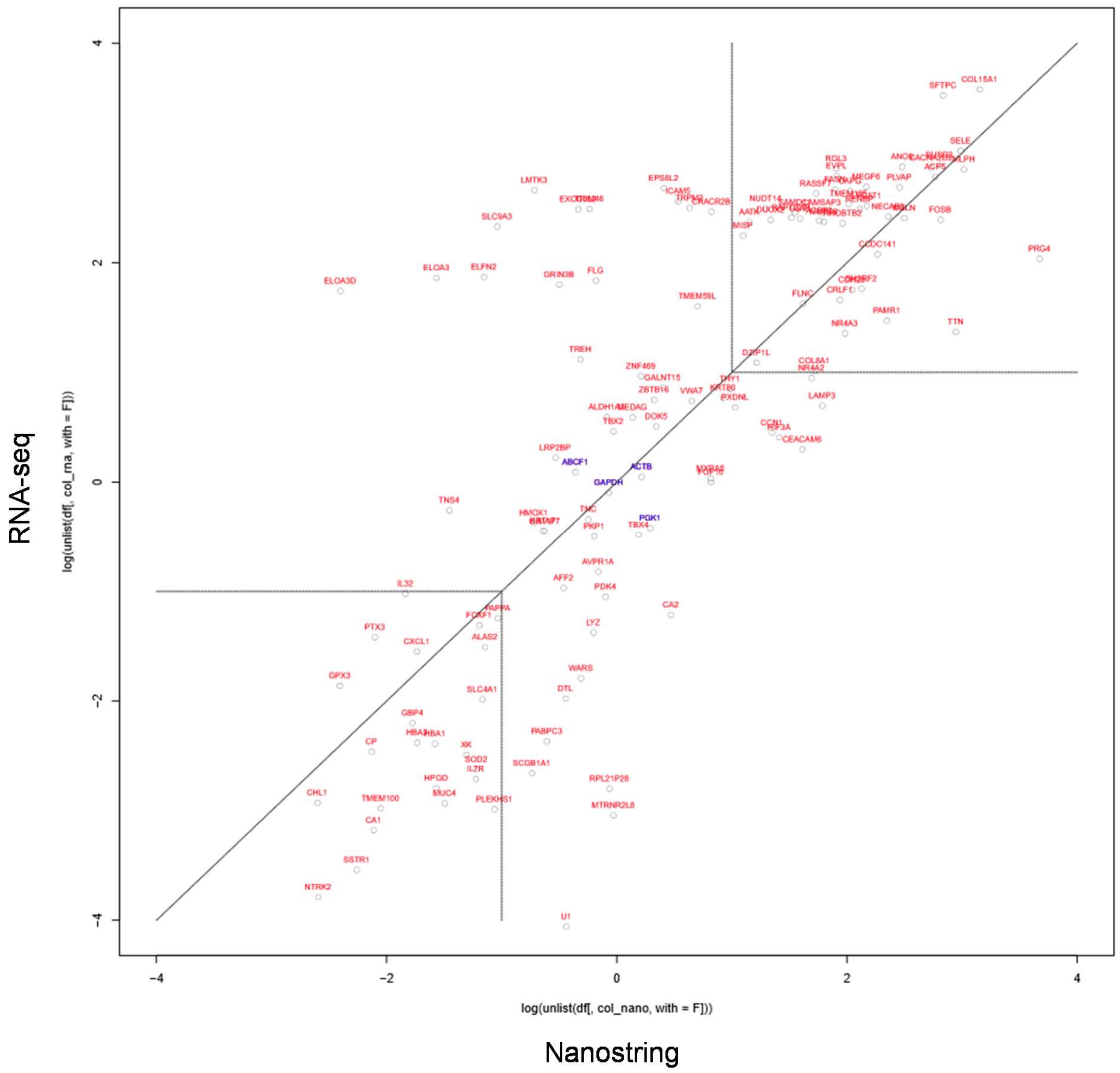

Additional file 12. Mutual comparison of RNA-seq and NanoString nCounter-determined changes in ACDMPV transcriptomes. Both methods detected relatively similar changes in expression levels of selected 120 genes.
