# Supplementary material for: Perturbation of semaphorin and VEGF signaling in ACDMPV lungs due to FOXF1 deficiency
Source: Respir Res. 2021 Jul 27;22:212. doi: 10.1186/s12931-021-01797-7 (PMC8314029; doi:10.1186/s12931-021-01797-7)
Supplement: Supplementary file 14 — Additional file 14. Bio-GPS graphs demonstrating similar fetal lung- and lung-specific expression pattern of TMEM100 and FOXF1. [file 12931_2021_1797_MOESM14_ESM.pdf]

TMEM100

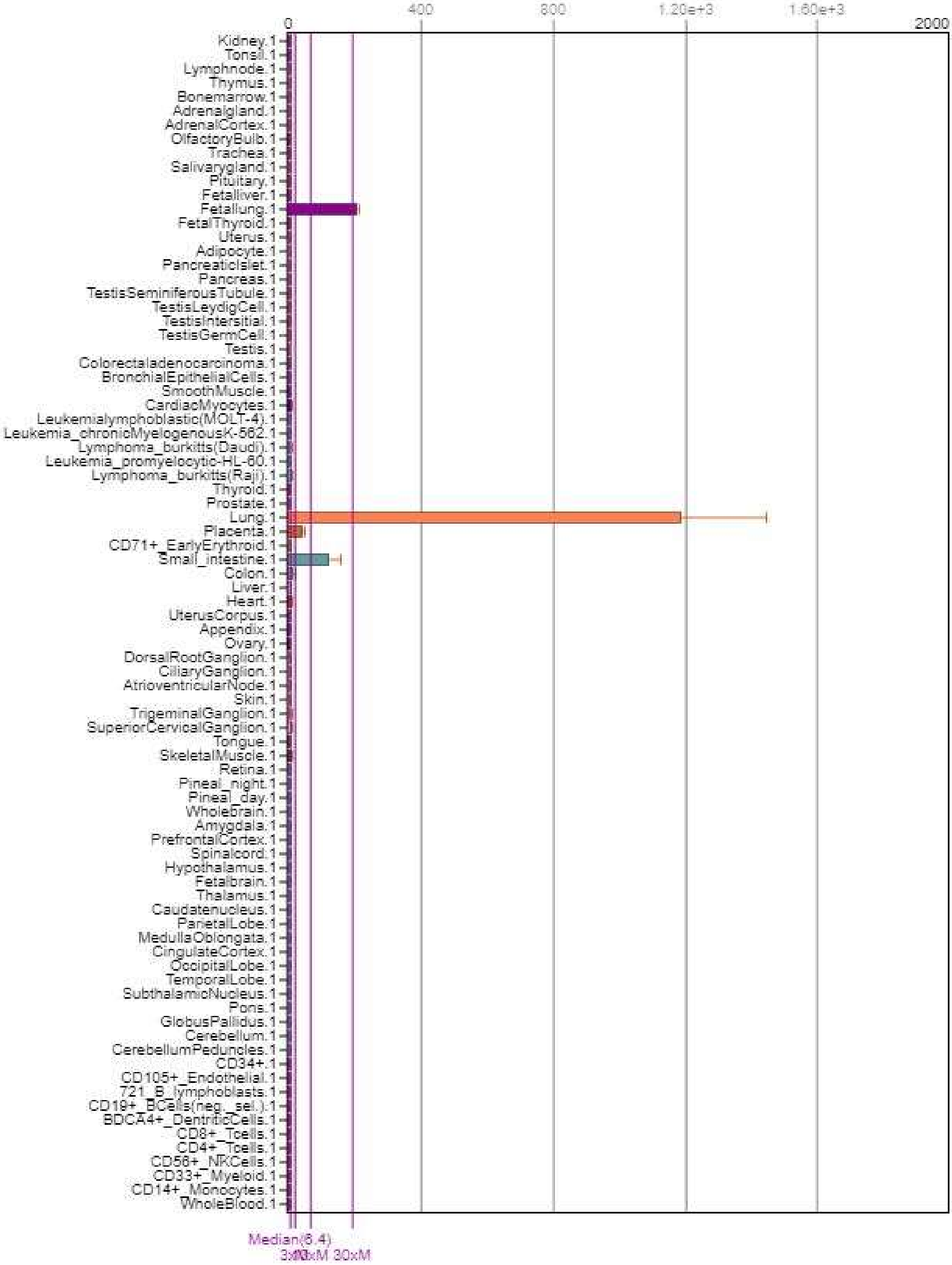
0
400
800
1.20e+3
1.60e+3
2000
Kidney.1
Tonsil.1
Lymphnode.1
Thymus.1
Bonemarrow.1
Adrenalgland.1
AdrenalCortex.1
OlfactoryBulb.1
Trachea.1
Salivarygland.1
Pituitary.1
Fetalliver.1
Fetallung.1
FetalThyroid.1
Uterus.1
Adipocyte.1
Pancreaticlslet.1
Pancreas.1
TestisSeminiferousTubule.1
TestisLeydigCell.1
TestisIntersitial.1
TestisGermCell.1
Testis.1
Colorectaladenocarcinoma.1
BronchialEpithelialCells.1
SmoothMuscle.1
CardiacMyocytes.1
Leukemialymphoblastic(MOLT-4).1
Leukemia_chronicMyelogenousK-562.1
Lymphoma_burkitts(Daudi).1
Leukemia_promyelocytic-HL-60.1
Lymphoma_burkitts(Raji).1
Thyroid.1
Prostate.1
Lung.1
Placenta.1
CD71+_EarlyErythroid.1
Small_intestine.1
Colon.1
Liver.1
Heart.1
UterusCorpus.1
Appendix.1
Ovary.1
DorsalRootGanglion.1
CiliaryGanglion.1
AtrioventricularNode.1
Skin.1
TrigeminalGanglion.1
SuperiorCervicalGanglion.1
Tongue.1
SkeletalMuscle.1
Retina.1
Pineal_night.1
Pineal_day.1
Wholebrain.1
Amygdala.1
PrefrontalCortex.1
Spinalcord.1
Hypothalamus.1
Fetalbrain.1
Thalamus.1
Caudatenucleus.1
ParietalLobe.1
MedullaOblongata.1
CingulateCortex.1
OccipitalLobe.1
TemporalLobe.1
SubthalamicNucleus.1
Pons.1
GlobusPallidus.1
Cerebellum.1
CerebellumPeduncles.1
CD34+.1
CD105+_Endothelial.1
721_B_lymphoblasts.1
CD19+_BCells(neg._sel.).1
BDCA4+_DentriticCells.1
CD8+_Tcells.1
CD4+_Tcells.1
CD56+_NKCells.1
CD33+_Myeloid.1
CD14+_Monocytes.1
WholeBlood.1
Median(6.4)
3xM
10xM
30xM

FOXF1

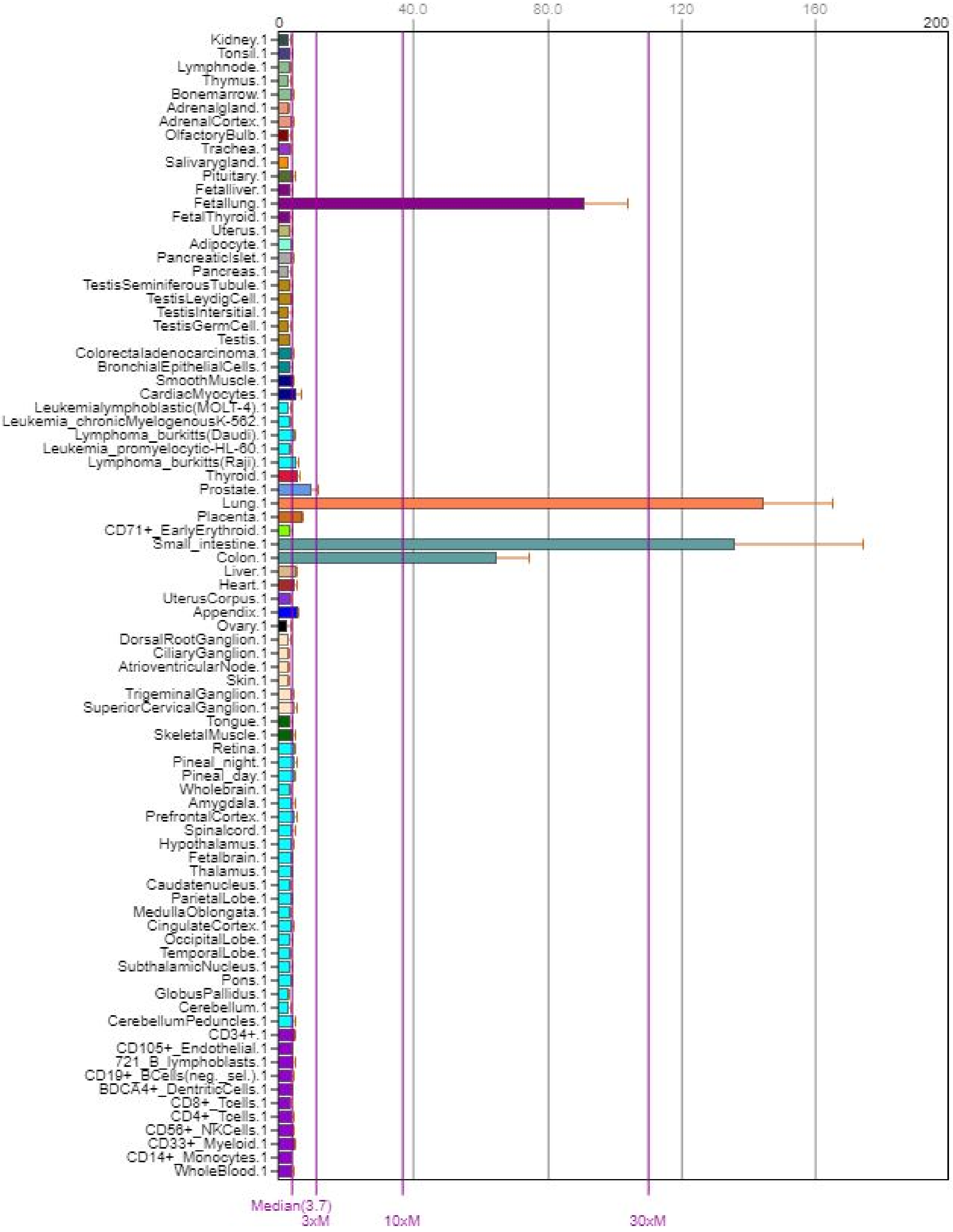
0
40.0
80.0
120
160
200
Kidney.1
Tonsil.1
Lymphnode.1
Thymus.1
Bonemarrow.1
Adrenalgland.1
AdrenalCortex.1
OlfactoryBulb.1
Trachea.1
Salivarygland.1
Pituitary.1
Fetalliver.1
Fetallung.1
FetalThyroid.1
Uterus.1
Adipocyte.1
PancreaticIslet.1
Pancreas.1
TestisSeminiferousTubule.1
TestisLeydigCell.1
TestisIntersitial.1
TestisGermCell.1
Testis.1
Colorectaladenocarcinoma.1
BronchialEpithelialCells.1
SmoothMuscle.1
CardiacMyocytes.1
Leukemialymphoblastic(MOLT-4).1
Leukemia_chronicMyelogenousK-562.1
Lymphoma_burkitts(Daudi).1
Leukemia_promyelocytic-HL-60.1
Lymphoma_burkitts(Raji).1
Thyroid.1
Prostate.1
Lung.1
Placenta.1
CD71+_EarlyErythroid.1
Small_intestine.1
Colon.1
Liver.1
Heart.1
UterusCorpus.1
Appendix.1
Ovary.1
DorsalRootGanglion.1
CiliaryGanglion.1
AtrioventricularNode.1
Skin.1
TrigeminalGanglion.1
SuperiorCervicalGanglion.1
Tongue.1
SkeletalMuscle.1
Retina.1
Pineal_night.1
Pineal_day.1
Wholebrain.1
Amygdala.1
PrefrontalCortex.1
Spinalcord.1
Hypothalamus.1
Fetalbrain.1
Thalamus.1
Caudatenucleus.1
ParietalLobe.1
MedullaOblongata.1
CingulateCortex.1
OccipitalLobe.1
TemporalLobe.1
SubthalamicNucleus.1
Pons.1
GlobusPallidus.1
Cerebellum.1
CerebellumPeduncles.1
CD34+.1
CD105+_Endothelial.1
721_B_lymphoblasts.1
CD19+_BCells(neg._sel.).1
BDCA4+_DentriticCells.1
CD8+_Tcells.1
CD4+_Tcells.1
CD56+_NKCells.1
CD33+_Myeloid.1
CD14+_Monocytes.1
WholeBlood.1
Median(3.7)
3xM
10xM
30xM

Additional file 14. Bio-GPS (http://biogps.org/#goto=welcome) graphs demonstrating similar fetal lung- and lung-specific expression pattern of *TMEM100* and *FOXF1*.
