# Supplementary material for: Perturbation of semaphorin and VEGF signaling in ACDMPV lungs due to FOXF1 deficiency
Source: Respir Res. 2021 Jul 27;22:212. doi: 10.1186/s12931-021-01797-7 (PMC8314029; doi:10.1186/s12931-021-01797-7)
Supplement: Supplementary file 3 — Additional file 3. CRISPR/Cas9 knockout of FENDRR in IMR-90 cell line in IMR-90 cell line. A Schematic representation of the FENDRR locus and CRISPR/Cas9-mediated FENDRR knockout strategy. Red box represents FENDRR exon 1; white box represents maximal 5’ extension of the exon 1, caused by alternative splicing, that is present in GENCODE transcript FENDRR-206 (ENST00000599749.5). F and R indicate primers (F: 5’-GAAAATGAAGGCGTCCGAGC, R: 5’-GCACATGCAAACCCGGTAAA) used in PCR amplification from normal (WT) and edited (KO) lung fibroblasts IMR-90. B Agarose gel electrophoresis of PCR products that verified promoter-exon 1 deletion (pe1∆). There was a minor heterogeneity with respect to the exact position of each of the deletion breakpoints. Sequence through the deletion junction and deletion coordinates refer to the predominant deletion (a small fraction of the deletions was 2 to 4 nt shorter). cCRE abbreviates FENDRR putative promoter (ENCODE). [file 12931_2021_1797_MOESM3_ESM.pdf]

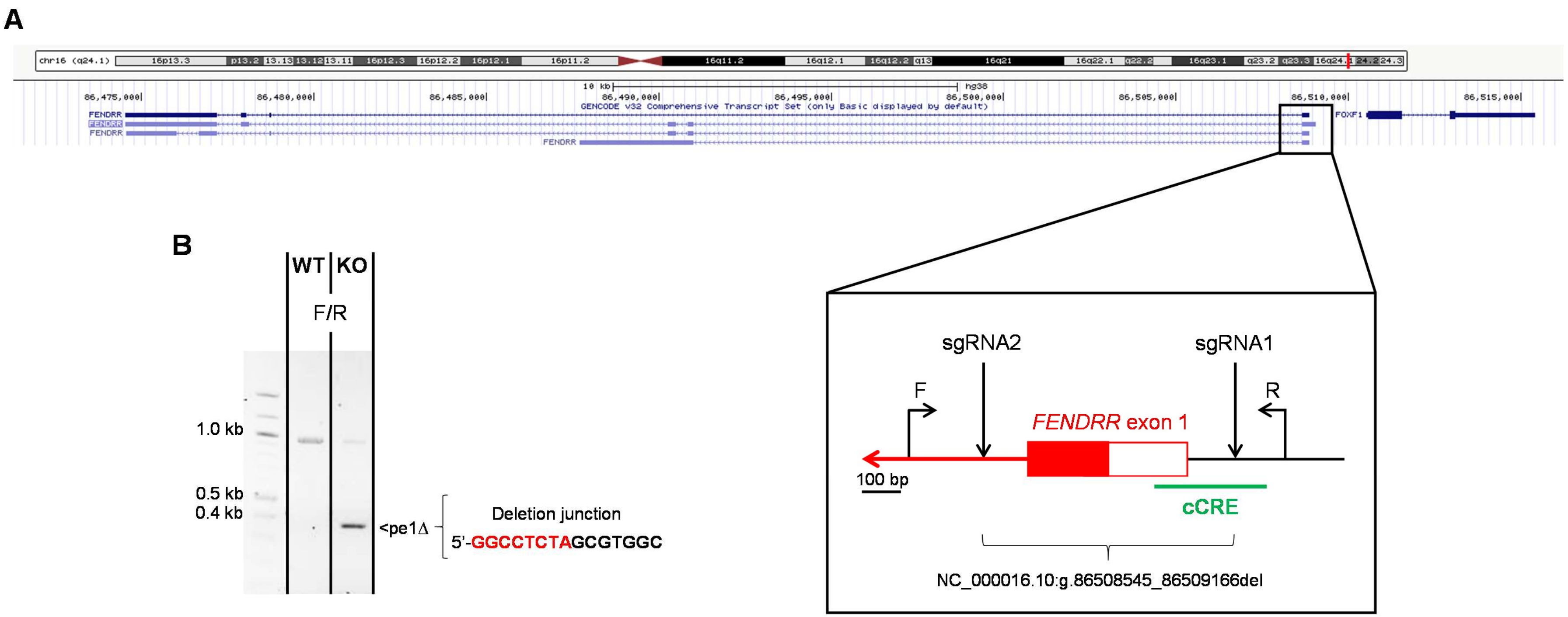

Additional file 3. CRISPR/Cas9 knockout of *FENDRR* in IMR-90 cell line in IMR-90 cell line. **A)** Schematic representation of the *FENDRR* locus and CRISPR/Cas9-mediated *FENDRR* knockout strategy. Red box represents *FENDRR* exon 1; white box represents maximal 5' extension of the exon 1, caused by alternative splicing, that is present in GENCODE transcript *FENDRR-206* (ENST00000599749.5). F and R indicate primers (F: 5'-GAAAATGAAGGCGTCCGAGC, R: 5'-GCACATGCAAACCCGGTAAA) used in PCR amplification from normal (WT) and edited (KO) lung fibroblasts IMR-90. **B)** Agarose gel electrophoresis of PCR products that verified promoter-exon 1 deletion (pe1Δ). There was a minor heterogeneity with respect to the exact position of each of the deletion breakpoints. Sequence through the deletion junction and deletion coordinates refer to the predominant deletion (a small fraction of the deletions was 2 to 4 nt shorter). cCRE abbreviates *FENDRR* putative promoter (ENCODE).
